# Assessment of Dried Blood Spots for Multi-Mycotoxin Biomarker Analysis in Pigs and Broiler Chickens

**DOI:** 10.3390/toxins11090541

**Published:** 2019-09-18

**Authors:** Marianne Lauwers, Siska Croubels, Siegrid De Baere, Milena Sevastiyanova, Eva Maria Romera Sierra, Ben Letor, Christos Gougoulias, Mathias Devreese

**Affiliations:** 1 Department of Pharmacology and Toxicology, Faculty of Veterinary Medicine, Ghent University, 9820 Merelbeke, Belgium; marianne.lauwers@ugent.be (M.L.); siska.croubels@ugent.be (S.C.); siegrid.debaere@ugent.be (S.D.B.); 2 Innovad Global, Postbaan 69, 2910 Essen, Belgium; m.sevastiyanova@innovad-global.com (M.S.); e.romera@innovad-global.com (E.M.R.S.); b.letor@innovad-global.com (B.L.); C.Gougoulias@innovad-global.com (C.G.)

**Keywords:** dried blood spots, pigs, broiler chickens, multi-mycotoxin analysis, toxicokinetic study, exposure assessment, biomarkers for exposure

## Abstract

Dried blood spots (DBSs), a micro-sampling technique whereby a drop of blood is collected on filter paper has multiple advantages over conventional blood sampling regarding the sampling itself, as well as transportation and storage. This is the first paper describing the development and validation of a method for the determination of 23 mycotoxins and phase I metabolites in DBSs from pigs and broiler chickens using liquid chromatography-tandem mass spectrometry (LC-MS/MS). The targeted mycotoxins belong to groups for which the occurrence in feed is regulated by the European Union, namely, aflatoxins, ochratoxin A and several *Fusarium* mycotoxins, and to two groups of unregulated mycotoxins, namely *Alternaria* mycotoxins and *Fusarium* mycotoxins (enniatins and beauvericin). The impact of blood haematocrit, DBS sampling volume and size of the analysed DBS disk on the validation results was assessed. No effects of variation in size of the analysed disk, haematocrit and spotted blood volume were observed for most mycotoxins, except for the aflatoxins and β-zearalanol (BZAL) at the lowest haematocrit (26%) level and for the enniatins (ENNs) at the lowest volume (40 µL). The developed method was transferred to an LC-high resolution mass spectrometry instrument to determine phase II metabolites. Then, the DBS technique was applied in a proof-of-concept toxicokinetic study including a comparison with LC-MS/MS data from plasma obtained with conventional venous blood sampling. A strong correlation (*r* > 0.947) was observed between plasma and DBS concentrations. Finally, DBSs were also applied in a pilot exposure assessment study to test their applicability under field conditions.

## 1. Introduction

The worldwide contamination of feed with mycotoxins is of major agro-economic importance. In addition to crop and feed loss and damage, these mycotoxins can have a large impact on animal health. Surveys show that mycotoxins occur in more than 70% of the tested feed samples and 38% of these samples contain multiple mycotoxins [1,2]. The co-contamination of several mycotoxins can result in additive, synergistic and antagonistic effects. Consequently, multi-method approaches are an asset in mycotoxin analysis [1].

The assessment of the exposure of pigs and broiler chickens to mycotoxins is mostly performed via feed analysis. However, this has major disadvantages such as the presence of hotspots and consequent inhomogeneous distributions of toxins, and the inability to determine individual exposure [3]. Therefore, the biomonitoring of mycotoxins in biological matrices using biomarkers for exposure is a growing field of interest. This has two major applications: exposure assessment studies to determine the exposure of animals to mycotoxins as well as toxicokinetic studies to evaluate the behaviour of mycotoxins in vivo and to test the efficacy of mycotoxin detoxifiers. Most analytical methods detect mycotoxins in urine or in plasma obtained via direct venepuncture [4,5,6,7,8]. More recently, micro-sampling techniques for quantitative analysis receive more and more attention due to their minimal invasiveness and the improved sensitivity of detection techniques such as liquid chromatography-tandem mass spectrometry (LC-MS/MS). The most commonly used micro-sampling technique in humans consists of the collection of a drop of whole blood obtained by a heel or finger prick on a dedicated filter paper. This so-called dried blood spot (DBS) sampling was first applied in human medicine for the detection of phenylketonuria in new-born infants [9]. The technique has major advantages compared to regular venous blood sampling. First, DBS collection is easy to perform, and the sampling volume is much smaller, which improves its use even in small animals. Furthermore, the storage and transport of the DBS poses less practical challenges and risks compared to plasma and urine due to increased analyte stability [10]. In that respect, DBSs are considered safe and non-contagious, in contrast to blood, plasma or serum, and this facilitates transport across the globe.

Cramer et al. [11] collected, for the first time, venous blood from human coffee and non-coffee drinkers in EDTA tubes and spotted 100 µL of this blood on Whatman^®^ 903 protein saver cards. Since *Aspergillus* species are identified as potent ochratoxin A (OTA) producers in coffee [12], OTA and its thermal degradation product 2′R-OTA were determined in DBS using LC-MS/MS. In the same year (2015), Riley et al. [13] evaluated the change of the sphinganine/sphingosine ratio as a biomarker for fumonisin B1 (FB1) exposure with the use of DBS. In 2016, Xue et al. [14] reported a good correlation of aflatoxin-lysine, a biomarker for chronic aflatoxin B1 (AFB1) exposure, between DBS and serum both in rats and humans (*r* of 0.996 and 0.784, respectively) [14].

All these studies involved single mycotoxin methods but, in reality, the co-occurrence of mycotoxins is far more common [1]. To date, only one study has analysed multiple mycotoxins in human DBS [15]. Osterech et al. [15] simultaneously quantified the following mycotoxins in DBS: aflatoxins (AFB1, AFB2, AFG1, AFG2, AFM1), deoxynivalenol (DON), DON-3-glucuronide (DON-3-GlcA), T2 toxin (T2), HT2 toxin (HT2), HT2-4-glucuronide (HT2-4-GlcA), FB1, OTA, *2′*R-*OTA*, ochratoxin alfa (OTα), 10-hydroxy-ochratoxin A (10-OH-OTA), citrinin (CIT), dihydrocitrinone (DH-CIT), zearalenone (ZEN), zearalanone (ZAN), altenuene (ALT), alternariol (AOH), alternariol monomethyl ether (AME), enniatins (ENNA, ENNA1, ENNB, ENNB1) and beauvericin (BEA). The limit of quantification (LOQ) ranged between 0.01 ng·mL^−1^ for ENNA1 and 5 ng·mL^−1^ for DON-3-GlcA and HT2, and the calibration ranged between 0.01 and 50 ng·mL^−1^, with the maximum concentration (Cmax) varying between 1 ng·mL^−1^ for the ENNs and 50 ng·mL^−1^ for T2, ZEN and metabolites. The authors also investigated the stability of the different components on the Whatman^®^ 903 protein saver cards and all components were stable (>90%), even after 24 weeks at −18 °C. 

Apart from their major advantages, DBSs could have some disadvantages as well. The exact quantification of the analytes of interest often remains problematic due to the haematocrit effect and the unknown volume of blood spotted exactly onto the filter paper. The haematocrit (volume percentage of red blood cells in whole blood) defines the viscosity of the blood. Variance in haematocrit causes differences in the diffusion of the blood over the filter paper, which can lead to a difference in analyte concentration. Moreover, the exact volume that is spotted might differ when directly applied to the card, as the size of a blood drop may vary between applications. Osteresch et al. [16] investigated the effect of haematocrit and spotted volume on the accuracy of OTA determination using Whatman^®^ 903 saver cards. No effect could be seen either for different haematocrit values (25%, 40%, and 55%) or for different blood drop volumes (75,100, and 125 µL). In addition, the dryness of the card and the type of card might influence the analysis. If the card is not totally dry, bacterial growth is possible, which may lead to the degradation of the analytes of interest. Different types of cards can have different pore sizes and thickness, impacting the blood spot size and volume. Finally, although differences in analyte concentration between venous and capillary blood can be observed [17], Osteresch et al. [16] did not observe such differences in OTA concentration [10,18]. Recently, efforts have been made to deal with such challenges, i.e., novel sampling devices are being produced and different strategies to cope with variation in haematocrit are being developed. These novelties include whole-spot analysis of volumetrically applied DBSs, the use of new sampling devices (e.g., volumetric absorptive microsampling (VAMS), which allows a constant sampling volume) and dried plasma spots, nicely summarised by [19].

DBSs are currently mostly applied in toxicology and therapeutic drug monitoring in human medicine [10,18]. Until now, no method is available for the detection of multiple mycotoxins in DBS from pigs and broiler chickens. In principle, such applications could be advantageous for inter alia toxicokinetic as well as exposure assessment studies with mycotoxins. The limited amount of blood needed facilitates application for smaller animal species and dramatically decreases the invasive character of blood collection, which, in turn, improves animal welfare. The ability of transport at room temperature and the non-contagious character of DBSs render them highly attractive in large scale studies. 

Therefore, the goal of this research was to develop and validate an LC-MS/MS method for the detection of EFSA-regulated AFB1, OTA, FB1, T2, ZEN and DON and the major phase I and II metabolites as well as non-regulated ENNs, BEA, AOH, AME and tenuazonic acid (TeA) [20,21,22] in DBS obtained from pigs and broiler chickens. In total, 23 mycotoxins and their phase I metabolites were included. Additionally, the impact of haematocrit differences, blood spot volumes applied on the filter paper, and size area (whole blood spot versus a distinct disk area) were evaluated. Finally, the DBS approach was evaluated both in (a) a proof-of-concept toxicokinetic study in pigs and broiler chickens with selected mycotoxins and (b) a pilot exposure assessment field trial in sows by comparing the results with data from jugular vein plasma analysis.

## 2. Results and Discussion

In this study, 23 mycotoxins and their phase I metabolites were selected for comparison of their concentration in plasma and DBS samples, based on the regulations of the European Union [20,21,23] and the occurrence data in feed [22,24]. These components were quantified in both matrices by LC-MS/MS. The phase II metabolites and interaction products were determined by liquid chromatography-high resolution mass spectrometry (LC-HRMS). 

### 2.1. Sample Extraction 

The optimal volume to cover a complete circle on the Whatman 903^®^ protein saver card was determined as 60 µL, as shown in Figure S1. Then, the blood (60 µL) was spiked with a mixture of 23 mycotoxins at a concentration of 10 ng·mL^−1^ each and spotted on the Whatman^®^ 903 protein saver card to optimize the extraction procedure. 

**Figure 1:**
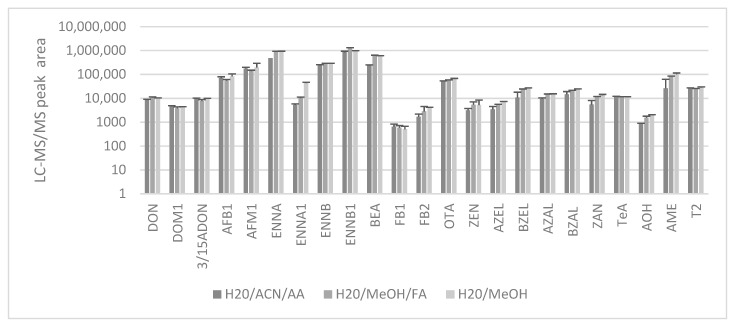
Evaluation of the analyte peak areas after LC-MS/MS analysis of DBS (60 µL) extracts which were redissolved in three different reconstitution solvents: water/acetonitrile(ACN)/acetic acid (AA) (95/5/0.1, *v*/*v*/*v*); water/methanol (MeOH)/formic acid (FA) (60/40/0.1, *v*/*v*/*v*) and water/MeOH(15/85, *v*/*v*). Individual mycotoxins were spiked in whole blood at a concentration of 10 ng·mL^−1^. The mean (*n* = 3) LC-MS/MS peak areas + standard deviation (SD) are shown in graph. Deoxynivalenol (DON), de-epoxy-deoxynivalenol (DOM1), 3/15-acetyl deoxynivalenol (3/15ADON), aflatoxin B1 (AFB1), aflatoxin M1 (AFM1), enniatin A (ENNA), enniatin A1 (ENNA1), enniatin B (ENNB), enniatin B1 (ENNB1), beauvericin (BEA), fumonisin B1 (FB1), fumonisin B2 (FB2), ochratoxin A (OTA), zearalenone (ZEN), α-zearalenol (AZEL), β-zearalenol (BZEL), α-zearalanol (AZAL), β-zearalanol (BZAL), zearalanone (ZAN), tenuazonic acid (TEA), alternariol (AOH), alternariol mono-methyl-ether (AME),T2 toxin (T2).

During initial experiments, an extraction solvent made of water/acetonitrile(ACN)/acetone (30/35/35, *v*/*v*/*v*) was evaluated as this mixture is used in literature for the extraction of mycotoxins from human DBS [11,15]. The extraction of pig DBS was performed in an ultrasonic bath for 30 min using 1 mL of this solvent mixture. After extraction, the filter paper was removed from the tube and the extraction solvent was dried under N_2_ gas at 45 ± 5 °C. Three different reconstitution solvents (n = 3 per condition) were evaluated: water/ACN/acetic acid (AA) (95/5/0.1, *v*/*v*/*v*); water/methanol (MeOH)/formic acid (FA) (60/40/0.1, *v*/*v*/*v*) and water/MeOH (15/85, *v*/*v*). The first two mixtures have previously been applied for the detection of multiple mycotoxins or OTA alone in human DBS [11,15]. The latter has been used by Lauwers et al. [4] in an LC-MS/MS method for multi-mycotoxin determination in pig and chicken plasma. The best results were obtained using MeOH instead of ACN in the reconstitution solvents (Figure 1). Next, calibration curves were made with the two MeOH containing reconstitution solvents and the lowest concentration achieved for each component as well as the peak shape were compared. The mixture of water/MeOH/FA (60/40/0.1, *v*/*v*/*v*) showed the best results for both parameters and was therefore chosen as the preferred solvent (data not shown). The same findings were also observed for DBS obtained from broiler chickens (data not shown). 

The DBSs were isolated from the filter paper in two ways prior to extraction: (a) by cutting out the entire spot using scissors and (b) by cutting out an 8 mm disk using a disposable biopsy punch. For pig DBSs, both techniques were used for method validation and results were compared. However, as the isolation of an 8 mm disk using a biopsy punch offers a more convenient and standardised approach, the validation of broiler chicken DBS was performed only with the 8 mm disks. 

### 2.2. Method Validation

The linearity expressed as a correlation coefficient (*r*) and the goodness-of-fit (g) is shown in Table 1 for pig DBSs and Table 2 for chicken DBSs and is the mean ± standard deviation of three curves across three different days of analysis. The *r* ranged between 0.991 and 0.999 for pigs and between 0.993 and 0.998 for broiler chickens. The g-value varied from 6% to 17% in pig and 8% to 19% in broiler chicken DBSs. Most of the calibration curves matched a linear model with a 1/x weighing factor, except for the ENNs and BEA which are best described by a quadratic model with a 1/x weighing factor. For most mycotoxins, the linearity ranged between 0.5 and 200 ng·mL^−1^. However, the limit of quantification (LOQ) and thus the lowest concentration in the calibration curve was set at 1 ng·mL^−1^ for ZEN, AZAL, BZEL, ZAN, DOM1 and AME in pigs and for ZEN, AZAL, BZAL, ZAN, AOH, T2, ENNA and FB1 in broiler chickens. In broiler chickens, AZEL had an LOQ of 4 ng·mL^−1^ and FB2 2 ng·mL^−1^ and in pigs AOH had an LOQ of 10 ng·mL^−1^. The results for the LOQ and corresponding LOD values are found in Table 1 and Table 2, respectively. 

The within-day and between-day precision and accuracy at a concentration of 10 and 100 ng·mL^−1^ met the requirements for all mycotoxins for both species. The results of the within-day and between-day precision and accuracy experiments can be found in Table 3 and Table 4, respectively.

The results for signal suppression or enhancement (SSE) after the extraction of DBSs from broiler chickens and pigs showed acceptable results (range 60–112%) for most components. Moreover, in pig DBSs, good extraction recovery rates were also observed. For some components, SSE was more pronounced and extraction recovery was rather low (<60%). However, for all mycotoxins, an adequate internal standard (IS) and matrix-matched calibration curves were used, resulting in validation results for accuracy and precision matching the acceptance criteria. The results for matrix effects and extraction recovery are shown in Table 2, Table 3 and Table 4.

### 2.3. Assessment of Dried Blood Spot Parameters

Apart from the standard validation parameters described above, other parameters were investigated, namely the difference between extracting the entire blood spot and only an 8 mm disk, the variation in spotted blood volume and the effect of haematocrit.

First, the correlation between the analysis of an 8 mm diameter disk and the entire blood spot was evaluated. Analysis of the entire spot required spotting of 60 µL whole blood on the Whatman^®^ 903 protein saver card and cutting out the complete spot with scissors to determine the analyte concentration. On the other hand, using an 8 mm biopsy punch, a standardized disk (8 mm diameter) could be excised precisely and uniformly from the protein saver card. This replaced the need of applying a pre-defined blood volume as long as the blood spot size exceeded the necessary 8 mm disk. In turn, this makes the DBS technique more suitable for routine analysis. 

First the linearity, LOQ and within-day and between-day precision and accuracy was determined for the 8 mm disk. Three calibration curves from pig DBSs were made on three different days, followed by the extraction of an 8 mm disk instead of the entire blood spot. The linearity of the curve was evaluated on the three days and LOQ was compared to the validation results of the entire spot. The linearity is expressed as a correlation coefficient (*r*) and the goodness-of-fit (g), which are shown in Table 5. The g-value ranged from 13% to 19% and the *r*-value varied between 0.991 and 0.998. The lowest concentration of the calibration curve for most components was 0.5 or 1 ng·mL^−1^, except for AOH (5 ng·mL^−1^) which is similar to the LOQ results obtained using the whole blood spot. The between-day precision and accuracy at concentrations of 10 and 100 ng·mL^−1^ and the within-day precision and accuracy at concentrations of 10 and 100 ng·mL^−1^ after the extraction of an 8 mm disk met the requirements, and is shown in Table 6. Consequently, the analysis of the proof-of-concept toxicokinetic study in pigs could be performed using both the extraction of the 8 mm disk and the entire blood spot. The obtained concentrations were plotted, and the correlation coefficient was determined. A good correlation (*r*) for AFB1 and DON was demonstrated, namely 0.998 and 0.946, respectively (Figure S2). 

In conclusion, the extraction of a standardized 8 mm diameter disk or the entire blood spot gave similar results for the mycotoxin concentrations in pig DBSs, which is in correspondence with Osteresch et al. for OTA determination in human DBSs [16]. As the use of 8 mm disks is more convenient than the entire blood spot, the method validation experiments and analysis of blood samples that were taken during a toxicokinetic study in broiler chickens were performed only using this 8 mm disk technique.

**Figure 2:**
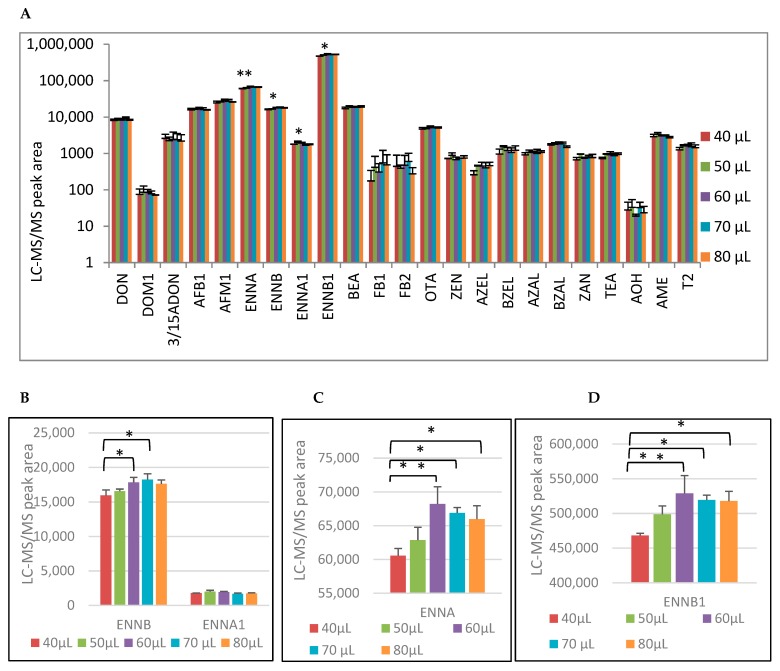
(**A**) Mean peak area + standard deviation (*n* = 3) of the different mycotoxins after extraction and LC-MS/MS analysis of different spotted pig blood volumes (respectively 40, 50, 60, 70, and 80 µL) on Whatman^®^ 903 protein saver cards. Mycotoxins with significant differences between the different volumes measured with a one-way-Anova are highlighted with an asterisk (ANOVA *p*-value of 0.05–0.01 *, *p*-value of 0.01–0.001 **). (**B**) The enlargement of (**A**) with a focus on ENNB and ENNA1. Post-hoc Bonferroni correction for pairwise comparison: *p*-value of 0.05–0.01 *, *p*-value of 0.01–0.001 **. (**C**) The enlargement of (**A**) with a focus on ENNA. Post-hoc Bonferroni correction for pairwise comparison: *p*-value of 0.05–0.01 *, *p*-value of 0.01–0.001 **. (**D**) The enlargement of (**A**) with a focus on ENNB1. Post-hoc Bonferroni correction for pairwise comparison: *p*-value of 0.05–0.01 *, *p*-value of 0.01–0.001 **.

Next, different volumes of pig blood were spotted on the Whatman^®^ 903 protein saver card. These ranged from under-filling (40–50 µL) to over-filling (70–80 µL) of the printed circle on the saver card. The DBSs were extracted using the 8 mm biopsy punch. The results are shown in Figure 2 as the mean LC-MS/MS peak areas + standard deviation (*n* = 3 per condition). A significant difference in measured peak areas was only observed for the ENNs. This was attributed to the peak areas measured in the spots with the lowest blood volume (40 µL). A one-way ANOVA showed a statistically significant difference between the different volumes for the ENNs: ENNA [F (4, 10) = 9.295, *p* = 0.002], ENNB [F (4,10) = 5.909 *p* = 0.01], ENNA1 [F (4,10) = 3.502 *p* = 0.049], ENNB1 [F (4,10) = 8193 *p* = 0.03]. A post-hoc Bonferroni correction revealed that the measured peak areas for some volumes were statistically significantly higher than for 40 µL. No statistically significant differences were observed between the other volumes. For ENNA, the peak areas corresponding with a spotted blood volume of 60 to 80 µL (on average 67,031 ± 1,931) were significantly higher (*p*-values of 0.004, 0.014 and 0.039, respectively) than the peak area in a 40 µL DBS sample (60,558 ± 1,063). For ENNB the peak areas in the 60 µL and 70 µL DBS (respectively 17,840 ± 736 and 18,241± 853) were significantly higher (*p*-values of 0.041 and 0.014, respectively) than the corresponding peak area in the 40 µL DBS (15,943 ± 793). Although ANOVA showed statistically significant differences for ENNA1, this could not be identified using post-hoc tests (Bonferroni). For ENNB1, the peak areas for the 60 to 80 µL DBS (on average 522,011 ± 15,919) were significantly higher (*p*-values of 0.005, 0.016 and 0.019, respectively) than the peak area for 40 µL (468,124 ± 3307). No statistically significant difference was observed for the remaining mycotoxins. 

In conclusion, only for the quantification of ENNs in pig DBS was it necessary to add at least 60 µL on the Whatman^®^ 903 protein saver card in order to obtain reliable results. These results are in agreement with Osteresch et al. [16], who did not see any influence of varying volume (75–125 µL), although the authors investigated only OTA. 

**Figure 3:**
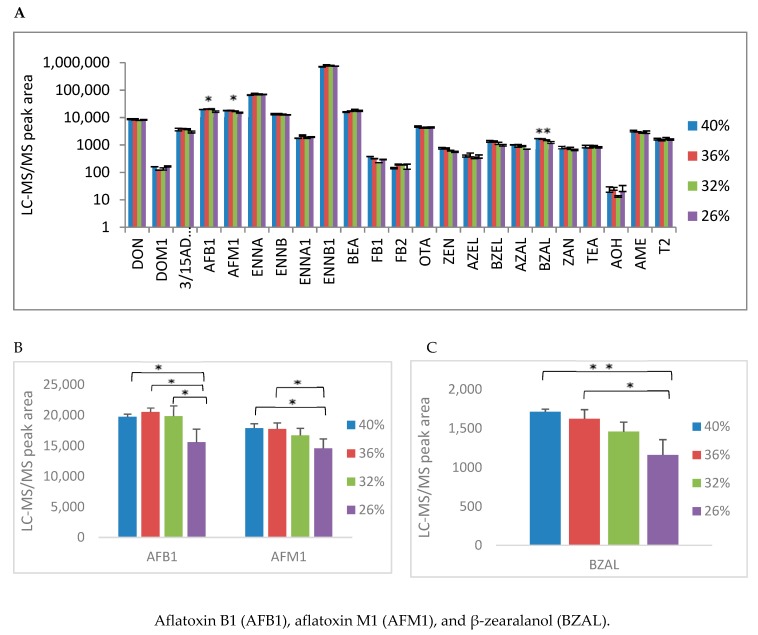
(**A**) Mean LC-MS/MS peak area + standard deviation of the different mycotoxins after extraction and analysis of dried pig blood spots with different haematocrit levels. The whole blood was spiked with relevant mycotoxins at a concentration level of 10 ng·mL^−1^ prior to spotting on Whatman^®^ protein saver cards. Mycotoxins with significant differences between the different haematocrit levels measured with a one-way Anova are highlighted with an asterisk (ANOVA *p*-value of 0.05–0.01 *, *p*-value of 0.01–0.001 **). (**B**) The enlargement of (**A**) with a focus on AFB1 and AFM1. Post-hoc Bonferroni correction for pairwise comparison: *p*-value of 0.05–0.01 *, *p*-value of 0.01–0.001 **. (**C**) The enlargement of (**A**) with a focus on BZAL. Post-hoc Bonferroni correction for pairwise comparison: *p*-value of 0.05–0.01 *, *p*-value of 0.01–0.001 **.

Finally, the haematocrit effect was measured by adding different volumes of blank plasma to whole blood. Normally, this value is approximately 35–40% and 25% is considered anaemic in pigs. The dilutions made were 89%, 80% and 66% of the normal haematocrit, thus corresponding with a haematocrit value of 36%, 32% and 26%. No significant difference could be observed in this range for most mycotoxins as shown in Figure 3. The lowest haematocrit only gave significant different results for BZAL and the aflatoxins (AFB1 and AFM1). A one-way ANOVA showed a statistically significant difference between the different haematocrit values for BZAL [F (3,8) = 10.529, *p* = 0.004], AFM1 [F (3,8) = 5.462, *p* = 0.024], and AFB1 [F (3,8) = 7.421, *p* = 0.011]. A post-hoc Bonferroni correction revealed that the measured peak areas of the analytes of interest after LC-MS/MS analysis were statistically significantly higher for the haematocrit values of 36% and 40% compared to a value of 26%. For BZAL, the LC-MS/MS peak areas at a haematocrit value of 36% and 40% (respectively 1624 ± 115 and 1713 ± 33) were significantly higher (*p*-values of 0.014 and 0.005, respectively) compared to the peak area at a haematocrit value of 26%. 

For AFM1, significantly higher peak areas (17,753 ± 977 and 17,876 ± 726, respectively) were observed at a haematocrit value of 36% and 40% (*p*-values of 0.036 and 0.030, respectively) compared to the peak area at a 26% haematocrit (14,606 ± 1490). For AFB1, the peak areas at haematocrit values of 32%, 36% and 40% (19,845 ± 1,703; 20,497 ± 700 and 19,727 ± 437, respectively) were significantly higher (*p*-values of 0.039, 0.018 and 0.045, respectively) than the peak area (15,562 ± 2155) at a haematocrit value of 26%. Therefore, reliable results can be obtained, except for anaemic animals.

In recent years, several collection devices have been developed which omit the variation in blood volume and haematocrit, with the most promising being the volumetric absorptive micro-sampling (VAMS) method. This technique allows to take the same volume of blood each time. Although very promising, sampling aberrant blood volumes still occurs. The two problems that occur most are the excess of blood by trapping blood on the handler and the abuse of the porous hydrophilic tip by greasy or poorly dried fingers and touching other materials, leading to loss or a higher volume of retained blood [18]. 

Since no or limited effects of varying volume and haematocrit have been observed on the measured mycotoxin peak areas in this study, DBSs can easily be used instead of VAMS, omitting the VAMS sampling problems and higher cost of the devices.

### 2.4. Biological Samples 

#### 2.4.1. Toxicokinetic Study

DON, ZEN and AFB1 were administered to pigs. The mycotoxin concentrations found in DBS were compared to the concentrations found in venous pig plasma, as shown in Figure S3. The correlation coefficients (r) were between 0.947 and 0.984 for DON (0.947), AFB1 (0.984), ZEN-GlcA (0.983) and DON-GlcA (0.952), which is considered a good correlation. 

Next, the plasma concentrations obtained after the administration of OTA, AFB1 and DON to broiler chickens were compared with the concentrations found in the DBS. Figure S4 shows the correlation between both sampling techniques. The correlation coefficient (*r*) for AFB1 is 0.974, for OTA 0.968 and for DON-sulphate (DON-S) 0.960. DON-S is considered the best biomarker after the exposure of broiler chickens to DON [25]

Both the toxicokinetic study in pigs and broiler chickens show a good correlation (*r* > 0.947) between concentrations in plasma and DBSs and DBSs can be considered a good alternative for plasma collection in toxicokinetic studies. Xue et al. [14] also observed a good correlation between serum AFB1-lysine concentrations in rats and the DBS concentrations after the administration of AFB1 in single and repeated doses. Moreover, in field conditions in Kenya, a positive correlation (*r*^2^ = 0.78) was found between AFB1-lysine in human serum and DBSs, although the number of positive samples using DBSs was lower [14]. Since AFB1 was only administered once in our study and the goal was to follow the concentration in time, AFB1 is a better biomarker than AFB1-lysine. The latter is mostly used after long-term exposure. To the authors’ knowledge, no studies were performed in which OTA, DON and ZEN were orally administered and DBSs determined. 

#### 2.4.2. Pilot Exposure Assessment Study

A pilot screening study was performed in five pig farms. In these farms, DBSs as well as plasma were collected from 20 sows. An example of chromatograms obtained after the analysis of plasma and DBSs of one animal is shown in Figure S5. In both biological matrices of this animal, DON and FB1 were found. The concentrations found in DBSs were 50 ng·mL^−1^ FB1 and 2 ng·mL^−1^ DON. On the other hand, the plasma concentrations were below the LOQ for DON and could not be determined for FB1, as this component was not validated in plasma. 

No correlation between the plasma and DBS concentrations could be found due to the low number of samples naturally contaminated with mycotoxins. Moreover, not the same mycotoxins were detected in each animal. 

## 3. Conclusions

A method was developed and validated for the determination of 23 mycotoxins and their phase I and II metabolites in dried blood spots of pigs and broiler chickens. No effect of variation in haematocrit and spotted blood volume were observed for most mycotoxins, except for the aflatoxins and BZAL at the lowest haematocrit (26%) level and for the ENNs at the lowest volume (40 µL). Moreover, both the extraction of a standardized 8 mm disk and the entire blood spot can be used to determine mycotoxins in DBS. The 8 mm disk method was preferred as this does not require a fixed volume. Finally, dried blood spots were applied in a toxicokinetic study and pilot exposure assessment study. A good correlation between plasma and dried blood spot data in the toxicokinetic study were observed. Therefore, dried blood spots appear to be an interesting micro-sampling technique, which can be used in future studies.

## 4. Methods and Materials

### 4.1. Chemicals, Products, and Reagents

The analytical standards of ZEN, T2, OTA, AFB1, AFM1, FB1, FB2, AOH, AME, TeA, DON, 3ADON, 15ADON, ENNA, ENNA1, ENNB1, ENNB and BEA were obtained from Fermentek (Jerusalem, Israel). ZAN, AZEL, BZEL, AZAL and BZAL were purchased from Sigma-Aldrich (Bornem, Belgium). DOM1 was obtained from Biopure (Tulln, Austria). Internal standards (IS) of ^13^C_15_-DON, ^13^C_24_-T2, ^13^C_18_-ZEN, ^13^C_20_-OTA, ^13^C_34_-FB1 and ^13^C_17_-AFB1 were purchased from Biopure. The IS ^13^C_6_^15^N-TeA was synthesized according to the method of Asam et al. [26], and ^15^N_3_-ENN B was synthesized according to the method of Hu and Rychlik [27]. All standards were stored according to the recommendations of the supplier.

Standard stock solutions (SSs) for ZEN, AZAL, BZAL, AZEL, BZEL, ZAN, DON, T2, AFB1, AFM1, AOH, AME, ENNs, BEA and FB1 were prepared in acetonitrile (ACN) at 100 µg·mL^−1^. Standard SS for OTA was prepared in ACN at 10 µg·mL^−1^. The standard SS of TeA was prepared in methanol (MeOH) at 100 µg·mL^−1^. The following standards were purchased as solutions: 3ADON (100 µg·mL^−1^ in ACN), 15ADON (100 µg·mL^−1^ in ACN) and DOM1 (50 µg·mL^−1^ in ACN). A standard SS of 10 µg·mL^−1^ in ACN was prepared for DOM1. A combined working solution of all analytical standards (without IS) at a concentration of 1 µg·mL^−1^ was prepared in ACN. Serial dilutions of the combined working solutions were prepared, yielding working solutions with concentrations of 100 and 10 ng·mL^−1^.

All ISs were obtained as solutions: ^13^C_15_-DON (25 µg·mL^−1^ in ACN), ^13^C_24_-T2 (25 µg·mL^−1^ in ACN), ^13^C_18_-ZEN (25 µg·mL^−1^ in ACN), ^13^C_20_-OTA (10 µg·mL^−1^ in ACN), ^13^C_34_-FB1 (25 µg·mL^−1^ in ACN/water) and ^13^C_17_-AFB1 (0.5 µg·mL^−1^ in ACN). Standard SSs of the synthesized internal standards were prepared at a concentration of 100 µg·mL^−1^ in MeOH for ^13^C_6_^15^N-TeA and 10 µg·mL^−1^ in ACN for ^15^N_3_-ENN B. The SSs were stored at ≤−15 °C. A combined working solution of all ISs (WSmix-IS) was prepared with a final concentration of 100 ng·mL^−1^ for all components, except ^13^C_17_-AFB1 (10 ng·mL^−1^). All working solutions were stored at ≤−15 °C.

Water, MeOH, ACN, ammonium formate, glacial acetic acid (AA) and formic acid (FA) were of LC-MS grade and were obtained from Biosolve (Valkenswaard, The Netherlands). Acetone and formic acid were of analytical grade and were purchased from VWR (Leuven, Belgium). Ostro^®^ 96 well plates were obtained from Waters (Milford, MA, USA). Whatman^®^ 903 protein saver cards were obtained from Novolab (Geraardsbergen, Belgium). Disposable biopsy punches were purchased from Kai Medical (Solingen, Germany).

DON and OTA for the animal trials were dissolved in analytical grade ethanol, and ZEN and AFB1 in dimethyl sulfoxide (DMSO) to obtain stock solutions of 10 mg·mL^−1^ DON, 5 mg·mL^−1^ OTA, 30 mg·mL^−1^ ZEN, and 10 mg·mL^−1^ AFB1. The solutions of DON, OTA and AFB1 were combined and mixed with HPLC-grade water to obtain a solution per broiler chicken corresponding to a dose of 0.5 mg·kg^−1^ BW DON, 0.25 mg·kg^−1^ BW OTA and 2 mg·kg^−1^BW AFB1. For pigs, the solutions of DON and ZEN were combined and mixed with HPLC-grade water to obtain a solution corresponding with a dose of 36 µg·kg^−1^ BW DON and 3 mg·kg^−1^ BW ZEN that was administered to the pigs. Next, the solution of AFB1 was mixed with HPLC-grade water to obtain a solution corresponding to a dose of 0.1 mg·kg^−1^ BW AFB1 to administer to the pigs.

### 4.2. Sample Preparation, LC-MS/MS and LC-HRMS Analysis

#### 4.2.1. Sample Preparation for DBS and Plasma

Venous blood was taken from the *vena jugularis* of healthy, untreated pigs and the *vena metatarsalis plantaris superficialis* of healthy, untreated broiler chickens using EDTA tubes as further explained under 4.5. First, different volumes of blood (100, 80 and 60 µL) were spotted on a Whatman^®^ 903 protein saver card to evaluate which volume was optimal to cover a whole circle on the card. Next, the blood (60 µL) was spiked with a mixture of 23 mycotoxins at a concentration of 10 ng·mL^−1^ and spotted on the Whatman^®^ 903 protein saver card to optimize the extraction procedure. 

The final extraction procedure was as follows. The entire DBS was cut out with scissors or a disk of 8 mm was punched out with a biopsy punch and added to a tube. Next, 1 mL of water/acetone/ACN (30/35/35, *v*/*v*/*v*) was added together with 20 µL of a 100 ng·mL^−1^ IS mix. The samples were extracted during 30 min in an ultrasonic bath. After extraction, the filter paper was removed, and the solvent was dried under a gentle N_2_-stream at 40 ± 5 °C. The dried material was reconstituted in 60 µL water/MeOH/FA (60/39.9/0.1, *v*/*v*/*v*).

Plasma sample pre-treatment was performed as previously described in Lauwers et al. [4]. In brief, 150 µL of chicken plasma was brought onto a well of an Ostro^®^-plate and 15 µL of a 100 ng·mL^−1^ (except ^13^C_17_-AFB1 10 ng·mL^−1^) IS combined working solution and 450 µL of ACN with 0.1% formic acid were added. The Ostro^®^ plate was brought under vacuum and the eluate was transferred to another tube and dried under a gentle N_2_-stream at 40 ± 5 °C. The dry residue was reconstituted in 150 µL of MeOH/water (85/15; *v*/*v*) and an aliquot of 5 µL was injected onto the chromatographic instruments (LC-MS/MS and LC-HRMS). 

To 250 µL of pig plasma, 20 µL of a 100 ng·mL^−1^ (except ^13^C_17_-AFB1 10 ng·mL^−1^) IS combined working solution and 750 µL of ACN with 0.1% formic acid were added, followed by vortex mixing (10 s) and centrifugation (8517× *g*, 10 min, 4 °C). The supernatant was collected and dried under a N2-stream at 40 ± 5 °C. The dried supernatant was reconstituted in 250 µL of MeOH/water (85/15; *v*/*v*), followed by vortex mixing. The reconstituted sample was transferred into an autosampler vial and an aliquot (5 µL) was injected onto the LC-MS/MS and LC-HRMS. 

#### 4.2.2. LC-MS/MS and LC-HRMS Analysis

The LC-MS/MS and LC-HRMS analysis was performed as previously described in Lauwers et al. [4]. An overview of the included mycotoxins and their phase I and II metabolites is given in Table S1. The structures of the parent mycotoxins and phase I metabolites measured using LC-MS/MS are shown in Table S2. 

### 4.3. DBS Method Validation

The LC-MS/MS method was validated according to a protocol previously described by De Baere et al. [8], using spiked blank whole blood spotted on a Whatman^®^ 903 protein saver card obtained from healthy, untreated animals fed a control diet (see Section 4.5.) The validation requirements are in compliance with the recommendations and guidelines defined by the European and international Community [28,29,30]. The following parameters were evaluated: linearity, within-day and between-day precision and accuracy, limit of quantification (LOQ), limit of detection (LOD), carry-over, specificity, extraction recovery and matrix effects.

#### 4.3.1. Linearity

Linearity was assessed by preparing three matrix-matched calibration curves with a concentration range of 0.5–200 ng·mL^−1^. The following concentration levels were included: 0, 0.5, 1, 2, 4, 5, 10, 30, 50, 100 and 200 ng·mL^−1^. The acceptance criteria for correlation coefficients (*r*) and the goodness-of-fit coefficients (g) were ≥0.99 and ≤20%, respectively [31]. 

#### 4.3.2. Precision and Accuracy

Within-day precision and accuracy were evaluated by analysing six blank samples spiked at medium (10 ng·mL^−1^) and high (100 ng·mL^−1^) concentration levels. Between-day precision and accuracy were determined by analysing in three-fold three quality control samples spiked at medium (10 ng·mL^−1^) and high (100 ng·mL^−1^) concentration level on three different days. The acceptance criteria for within-day and between-day accuracy were as follows: −50% to +20%, −30% to +10% and −20 to +10% for concentrations of ≤1 ng·mL^−1^, 1–10 and ≥10 ng·mL^−1^, respectively. For the within-day precision, the relative standard deviation (RSD%) had to be lower than the maximum relative standard deviation (RSDmax), which was <25%, and <15% for concentrations ≥1 to <10 ng·mL^−1^ and ≥10 to <100 ng·mL^−1^, respectively [30]. For the between-day precision, the RSD% had to be lower than the RSDmax, which was defined by the Horwitz equation: RSDmax = 2(1–0.5 log Concentration (g·mL^−1^)). The RSDmax was 22.6% and 32% for the respective concentrations of 100 and 10 ng·mL^−1^ [28,29].

#### 4.3.3. Limit of Quantification (LOQ)

The LOQ was the lowest concentration of the analyte for which the method was validated with an acceptable accuracy and precision according to the guidelines described above. The LOQ was also the lowest concentration of the calibration curves. The LOQ was determined by analysing different concentrations spiked in six-fold on the same day.

#### 4.3.4. Limit of Detection (LOD)

The LOD is the lowest concentration that gives a signal that significantly differs from the signal of a blank sample. The LOD was determined by calculating the analyte concentration that corresponds with a S/N ratio of 3/1 (signal-noise-ratio in a sample at the retention time of the analyte), based on the S/N ratio of the analyte in the LOQ samples. 

#### 4.3.5. Carry-over

Carry-over was assessed by analysing the solvent (mixture of MeOH/water/FA (39.9/60/0.1; *v*/*v*/*v*)) directly after the highest calibrator (200 ng·mL^−1^). The response of a peak that elutes eventually at the same retention time as the analyte should be no more than 20% of the mean peak area of the analyte in the LOQ samples. The peak area of the IS should be no more than 5% of the average peak area of the IS in the calibrator samples and quality control (QC) samples.

#### 4.3.6. Specificity

The specificity of the method was evaluated with respect to the interference of endogenous components. Hence, a blank sample was analysed. If a peak is detected in the blank sample at the same retention time as the analyte, the response of this peak should be no more than 20% of the mean peak area of the analyte in the LOQ samples. 

#### 4.3.7. Extraction Recovery and Matrix Effects

The extraction recovery and matrix effects of the method were calculated according to the method of Matuszewski et al. [32]. Therefore, three types of samples were prepared. The first set of samples were matrix-matched and prepared by spiking blank samples before extraction (=Spiked). The second set of samples consisted of matrix-matched blank samples that were spiked after extraction (=SpikedExtract). The third series of samples were standard solutions (=Standard). All samples were spiked at 10 ng·mL^−1^ and were made in triplicate. 

The peak areas of the analytes of interest in these samples were compared to calculate the signal suppression or enhancement (SSE) due to matrix effects and the recovery of the extraction step (Re), according to the following formulas:

SSE = 100 × (Area SpikedExtract/Area Standard)
(1)

Re = 100 × (Area Spiked/Area SpikedExtract)
(2)

### 4.4. Assessment of DBS Parameters

Blank whole blood samples were obtained from healthy, untreated pigs and broiler chickens as further explained under Section 4.5. These blood samples were used to analyse the difference between analysing the whole spot versus a distinct 8 mm disk, the impact of different sampling volumes and the differences in haematocrit, as these parameters can have an impact on the quantitative analysis of selected analytes in DBS.

#### 4.4.1. Impact of Extracting an 8 mm Disk vs Entire Spot

To compare the two methods, first the validation parameter linearity, between-day and within-day precision and accuracy and the LOQ were determined for the extraction using an 8 mm disk. The validation parameters should meet the criteria previously determined. Next, the results of both the 8 mm disk and cutting out the entire DBS were compared. Finally, the concentrations obtained in a toxicokinetic study using both techniques were compared.

#### 4.4.2. Impact of Blood Volume Spotted

The blank whole blood samples were spiked at a concentration level of 10 ng·mL^−1^. Next, different volumes (40, 50, 60, 70 and 80 µL) were spotted in triplicate on the Whatman^®^ 903 protein saver cards. The samples were dried overnight and stored at −18 °C until analysis. The DBSs were extracted using the 8 mm biopsy punch. A calibration curve was made by spotting 60 µL of whole blood with different concentrations on the Whatman^®^ 903 protein saver cards, as this volume is in accordance with the fully saturated circle. The calibration curve was also extracted with the 8 mm disk. 

#### 4.4.3. Impact of Haematocrit Level

The blank whole blood samples were diluted with plasma, followed by spiking at a concentration level of 10 ng·mL^−1^. The normal haematocrit value is between 35% and 40% and a value below 25% is considered anaemic in pigs [33]. A volume of 60 µL of an undiluted sample and three dilutions (89%, 80% and 66%) were spotted in triplicate, corresponding with an estimated haematocrit value of 40%, 36%, 32% and 26%. The samples were dried overnight and stored at −18 °C until analysis. The DBSs were extracted using the 8 mm biopsy punch. A calibration curve was made by spotting 60 µL of undiluted blood with different concentrations on the saver cards as this volume is in accordance with the fully saturated circle.

### 4.5. Toxicokinetic Trial

Incurred plasma samples were obtained from two 12 h fasted hybrid pigs (6 weeks of age, 9.93 ± 0.89 kg BW), dosed with a single oral (intragastric) bolus of DON (36 µg·kg^−1^ BW) and ZEN (3 mg·kg^−1^ BW) (experiment 1), from two 12 h fasted hybrid pigs (4 weeks of age, 9.31 ± 0.82 kg BW) dosed with AFB1 (0.1 mg·kg^−1^ BW AFB1) (experiment 2), and from two 12 h fasted broiler chickens (Ross 308, 3 weeks of age, 1.04 ± 0.09 kg) administered AFB1 (2 mg·kg^−1^ BW), DON (0.5 mg·kg^−1^ BW) and OTA (0.25 mg·kg^−1^ BW) (experiment 3). All mycotoxin doses were administered as a single oral bolus (by gavage). For DON, this dose was in agreement with the EU legislation in feed. The maximum guidance level in pig feed is set at 0.9 mg·kg^−1^ DON [21]. Pigs of this age category consume on average 40 g feed kg^−1^ BW/day. This resulted in the administration of 36 μg DON·kg^−1^ BW as described in [34]. For broiler chickens, the EU regulations set the maximum guidance level at 5 mg·kg^−1^ feed [21]. Broilers (± 1 kg BW) consume on average 100 g feed·kg^−1^ BW/day. This resulted in the administration of 0.5 mg DON·kg^−1^ BW as described in [34]. For ZEN, OTA and AFB1 in pigs and AFB1 and OTA in broiler chickens, the administered doses corresponded to the doses previously administered in toxicokinetic studies and studies to determine the efficacy of mycotoxin detoxifiers [25,35]. At these mycotoxin inclusion rates, no clinical symptoms were observed in this study or in previous studies.

Blood was sampled before administration (0 min) and at 5, 10, 20, 30, 45, 60, 90 and 120 min, as well as at 3, 4, 6, 8, 10, 12 and 24 h after the administration of mycotoxins. Blood was collected via the *vena jugularis* (pigs) and the *vena metatarsalis plantaris superficialis* (broiler chickens) in EDTA tubes using a Venoject^®^ system and centrifuged (2851× *g*, 10 min, 4 °C) to obtain plasma, which was stored at ≤−15 °C until analysis. Dried blood spots were made from the obtained blood samples by taking 60 µL of whole blood and spotting on the Whatman^®^ 903 protein saver cards (*n* = 2 per sample). The DBSs were stored at −18 °C after overnight drying. 

A multi-mycotoxin LC-MS/MS analysis of the pigs and chicken feed (Primoris, Zwijnaarde, Belgium) showed only low amounts of DON (respectively, 139, 100 and 140 µg·kg^−1^) and ZEN (respectively, 12, 18 and 20 µg·kg^−1^) for experiment 1 to 3. The feed conformed to the EU legislation, since these amounts were below the guidance values of the European Commission [21]. Blank plasma and whole blood samples were obtained from 2 pigs and broiler chickens on this control diet. The blank samples were used for the preparation of matrix-matched calibrator, quality control and validation samples and for the assessment of dried blood spot parameters.

The animal trial was approved by the ethical committee of the Faculty of Veterinary Medicine and the Faculty of Bioscience Engineering of Ghent University (EC2017/05 and EC2017/12) respectively on 30 March 2017 and 20 March 2017.

### 4.6. Pilot Exposure Assessment Study

A pilot exposure assessment study (*n* = 5 pig farms) was performed in Lleida region, Spain, to monitor the exposure of pigs to mycotoxins and to demonstrate the applicability of the developed method in the field. Therefore, farms with postpartum problems that might be related to mycotoxins and where mycotoxins were found in feed were selected. Blood of 12 sows on each farm (6 lactating sows and 6 gestation sows) was collected approximately 30 min after feeding. The blood was drawn from the vena jugularis, collected in EDTA tubes, and centrifuged (2851× *g*, 10 min, 4 °C) to obtain plasma. DBS were obtained from the ear of the sow and directly spotted on the Whatman^®^ 903 protein saver card. The card was dried overnight. Next, both plasma and DBS were stored at −18 °C until analysis. The animal trial was approved by the ethical committee of the Faculty of Veterinary Medicine and the Faculty of Bioscience Engineering of Ghent University (EC2017/115).

### 4.7. Statistical Analysis

The LC-MS/MS peak area (*n* = 3) of the mycotoxins to assess the impact of different spotted volumes and haematocrit values were expressed as the mean ± standard deviation and compared using an analysis of variance (ANOVA) followed by a post-hoc Bonferroni correction for multiple comparison. Before performing the one-way ANOVA, the normality and homogeneity of variance was checked. The level of significance was set at 0.05. The Pearson correlation coefficient was used to measure the strength of the association between pairs of variables. All analyses were conducted using SPSS 24 (IBM, Chicago, IL, USA).

## Figures and Tables

**Table 1:** Validation results for linearity shown as the mean ± standard deviation of three curves across three different days of analysis (linear range, correlation coefficient (*r*) and goodness-of-fit coefficient (g)), limit of quantification (LOQ) and limit of detection (LOD) of 23 mycotoxins in dried blood spots of pig whole blood.

Analyte	Linearity (*n* = 3 Different Days)			LOQ (ng·mL^−1^)	LOD (ng·mL^−1^)
	Linear Range (ng·mL^−1^)	*r* ± SD	g (%) ± SD		
ZEN	1–200	0.999 ± 0.001	15 ± 2	1.0	0.09
AZEL	0.5–200	0.998 ± 0.000	14 ± 7	0.5	0.20
AZAL	1–200	0.998 ± 0.001	11 ± 4	1.0	0.38
BZAL	0.5–200	0.997 ± 0.001	16 ± 7	0.5	0.11
BZEL	1–200	0.994 ± 0.004	16 ± 2	1.0	0.21
ZAN	1–200	0.996 ± 0.004	10 ± 6	1.0	0.10
TeA	0.5–200	0.996 ± 0.003	16 ± 2	0.5	0.04
AOH	10–200	0.994 ± 0.005	14 ± 8	10	0.74
AME	1–200	0.993 ± 0.002	10 ± 2	1.0	0.01
DON	0.5–200	0.994 ± 0.003	17 ± 2	0.5	0.11
DOM1	1–200	0.997 ± 0.002	10 ± 2	1.0	0.23
3/15ADON	0.5–200	0.994 ± 0.002	14 ± 2	0.5	0.06
T2	0.5–200	0.996 ± 0.004	14 ± 2	0.5	0.01
AFB1	0.5–200	0.994 ± 0.002	16 ± 3	0.5	0.001
AFM1	0.5–200	0.991 ± 0.002	11 ± 1	0.5	0.01
OTA	0.5–200	0.995 ± 0.003	12 ± 5	0.5	0.01
ENN A1	0.5–200	0.997 ± 0.002	14 ± 5	0.5	0.05
ENNA	0.5–100	0.998 ± 0.001	9 ± 6	0.5	0.01
ENNB	0.5–100	0.999 ± 0.000	6 ± 4	0.5	0.001
ENNB1	0.5–100	0.998 ± 0.001	10 ± 2	0.5	0.001
BEA	0.5–200	0.997 ± 0.003	13 ± 5	0.5	0.02
FB2	0.5–200	0.996 ± 0.002	17 ± 1	0.5	0.35
FB1	1–200	0.994 ± 0.004	15 ± 3	0.5	0.23

Note: SD, standard deviation; acceptance criteria: r ≥ 0.990 and g ≤ 20%. zearalenone (ZEN), α-zearalenol (AZEL), α-zearalanol (AZAL), β-zearalanol (BZAL), β-zearalenol (BZEL), zearalanone (ZAN), tenuazonic acid (TEA), alternariol (AOH), alternariol mono-methyl-ether (AME), Deoxynivalenol (DON), de-epoxy-deoxynivalenol (DOM1), 3/15-acetyl deoxynivalenol (3/15ADON), T2 toxin (T2), aflatoxin B1 (AFB1), aflatoxin M1 (AFM1), ochratoxin A (OTA), enniatin A1 (ENNA1), enniatin A (ENNA), enniatin B1 (ENNB1), enniatin B (ENNB), beauvericin (BEA), fumonisin B2 (FB2), fumonisin B1 (FB1).

**Table 2:** Validation results for linearity shown as the mean ± standard deviation of three curves across three different days of analysis (linear range, correlation coefficient (*r*) and goodness-of-fit coefficient (g)), limit of quantification (LOQ) and limit of detection (LOD) of 23 mycotoxins in dried blood spots of broiler chicken whole blood.

Analyte	Linearity (*n* = 3 Different Days)			LOQ (ng·mL^−1^)	LOD (ng·mL^−1^)
	Linear Range (ng·mL^−1^)	*r* ± SD	g ± SD		
ZEN	1–200	0.997 ± 0.001	17 ± 3	1.0	0.12
AZEL	4–200	0.997 ± 0.002	8 ± 3	4.0	1.10
AZAL	1–200	0.997 ± 0.002	16 ± 6	1.0	0.15
BZAL	1–200	0.996 ± 0.003	18 ± 2	1.0	0.27
BZEL	0.5–200	0.996 ± 0.003	16 ± 2	0.5	0.1
ZAN	1–200	0.997 ± 0.001	13 ± 5	1.0	0.21
TeA	0.5–200	0.998 ± 0.001	12 ± 6	0.5	0.001
AOH	1–200	0.996 ± 0.002	17 ± 3	1.0	0.02
AME	0.5–200	0.997 ± 0.001	17 ± 4	0.5	0.001
DON	0.5–200	0.997 ± 0.000	15 ± 1	0.5	0.18
DOM1	0.5–200	0.998 ± 0.001	17 ± 1	0.5	0.16
3/15ADON	0.5–200	0.995 ± 0.003	16 ± 4	0.5	0.09
T2	1–200	0.998 ± 0.000	13 ± 5	1.0	0.03
AFB1	0.5–200	0.997 ± 0.001	15 ± 4	0.5	0.01
AFM1	0.5–200	0.997 ± 0.001	15 ± 2	0.5	0.01
OTA	0.5–200	0.998 ± 0.001	19 ± 1	0.5	0.05
ENN A1	0.5–200	0.993 ± 0.002	14 ± 5	0.5	0.04
ENNA	1–200	0.995 ± 0.003	16 ± 2	1.0	0.11
ENNB	0.5–200	0.994 ± 0.001	12 ± 1	0.5	0.07
ENNB1	0.5–200	0.998 ± 0.002	12 ± 3	0.5	0.001
BEA	0.5–200	0.996 ± 0.000	15 ± 4	0.5	0.07
FB2	2–200	0.997 ± 0.001	16 ± 3	1.0	0.96
FB1	1–200	0.995 ± 0.001	15 ± 3	2.0	1.87

Note: SD, standard deviation; acceptance criteria: *r* ≥ 0.990 and g ≤ 20%.

**Table 3:** Validation results for within-day and between-day precision and accuracy, matrix effects (signal suppression or enhancement, SSE) and extraction recovery (Re) of 23 mycotoxins in dried blood spots of pig whole blood.

Analyte	Within-day Precision and Accuracy (*n* = 6)				Between-day Precision and Accuracy (*n* = 3 × 3)				SSE (%)	Re (%)
	Theoretical Concentration 10 ng·mL^−1^		Theoretical Concentration 100 ng·mL^−1^		Theoretical Concentration 10 ng·mL^−1^		Theoretical Concentration 100 ng·mL^−1^			
	Precision (RSD %)	Accuracy (%)	Precision (RSD %)	Accuracy (%)	Precision (RSD %)	Accuracy (%)	Precision (RSD %)	Accuracy (%)		
ZEN	13	−2.3	8	2.5	11	−0.6	8	4.1	46	89
AZEL	6	8.8	7	9.8	15	−5.8	8	2.5	109	52
AZAL	12	−6.1	4	9.4	14	−2.1	12	3.4	61	78
BZAL	10	−0.6	8	4.9	10	2.3	12	2.2	72	77
BZEL	12	0.7	4	−15.8	16	−4.9	13	−5.1	94	66
ZAN	7	3.9	9	4.3	8	5.0	9	7.1	77	85
TeA	4	−0.9	2	0.3	9	2.4	6	5.3	78	129
AOH	12	−19.7	8	−12.6	10	−21.3	11	−4.8	35	61
AME	9	−6.2	8	−6.8	28	−8.6	16	−8.2	32	55
DON	9	+8.6	8	2.6	8	5.9	7	4.6	38	117
DOM1	8	−1.3	9	4.4	7	−1.3	12	2.5	9	105
3/15ADON	8	6.9	3	6.7	12	4.7	9	9.1	48	74
T2	13	−2.8	10	6.9	11	0.7	8	9.4	65	96
AFB1	9	−13.8	6	−9.5	13	−4.1	13	2.6	48	66
AFM1	12	7.2	10	5.8	12	4.9	7	7.6	77	64
OTA	5	−8.3	4	−2.5	13	0.3	10	6.6	70	95
ENN A1	5	−7.0	7	−1.4	10	−3.9	7	−5.9	56	88
ENNA	2	−14.5	2	−14.5	10	−8.6	16	−12.5	99	96
ENNB	10	−2.5	10	1.2	15	−9.3	20	−16.4	57	106
ENNB1	2	−1.0	8	−5.1	9	−3.5	10	0.0	82	99
BEA	8	−14.5	9	−13.0	9	−11.5	20	−7.9	79	80
FB2	14	−6.7	5	−17.6	13	−3.4	20	−4.9	54	64
FB1	7	−7.3	1	−10.5	12	−2.5	12	−1.5	55	67

Note: acceptance criteria: accuracy ≥10 ng·mL^−1^: −20% to +10%. Within-day precision: relative standard deviation (RSD%) < RSDmax with RSDmax for ≥10 to <100 ng·mL^−1^: <15%. Between-day precision: the RSD% < RSDmax with RSDmax 22.6% and 32% for the respective concentrations of 100 and 10 ng·mL^−1^, respectively

**Table 4:** Validation results for within-day and between-day precision and accuracy, matrix effects (signal suppression or enhancement, SSE) and extraction recovery (Re) of 23 mycotoxins in dried blood spots of broiler chicken whole blood.

Analyte	Within-day Precision and Accuracy (*n* = 6)				Between-day Precision and Accuracy (*n* = 3 × 3)				SSE (%)	Re(%)
	Theoretical Concentration 10 ng·mL^−1^		Theoretical Concentration 100 ng·mL^−1^		Theoretical Concentration 10 ng·mL^−1^		Theoretical Concentration 100 ng·mL^−1^			
	Precision (RSD %)	Accuracy (%)	Precision (RSD %)	Accuracy (%)	Precision (RSD %)	Accuracy (%)	Precision (RSD %)	Accuracy (%)		
ZEN	10	−5.8	5	4.8	12	−6.8	7	−2.1	70	18
AZEL	11	−5.3	4	8.6	17	−11.0	12	2.1	100	16
AZAL	6	−2.6	4	5.0	12	−9.6	10	1.1	99	18
BZAL	8	−11.9	5	−2.0	13	−17.8	9	−3.4	94	21
BZEL	7	−8.4	6	−11.0	11	−11.7	14	−11.0	81	17
ZAN	7	0.0	7	−5.0	12	−3.3	10	−5.4	95	21
TeA	9	−3.5	5	−0.2	10	−3.1	6	0.6	104	19
AOH	12	−8.9	4	6.0	12	−12.2.	6	5.2	95	19
AME	13	−1.7	8	−6.3	13	−7.7	16	−3.2	86	15
DON	3	5.8	5	1.5	5	4.8	7	−1.4	44	88
DOM1	11	−4.8	8	−3.3	10	−5.6	7	−4.3	64	28
3/15ADON	2	−10.5	6	2.1	13	−9.3	12	−3.7	69	73
T2	5	−0.2	5	6.9	6	−1.6	5	7.0	85	37
AFB1	7	−3.0	4	6.1	18	−7.5	5	4.9	71	39
AFM1	8	−3.3	5	1.3	9	−8.8	6	2.7	91	38
OTA	9	−18.3	5	−2.1	11	−15.7	5	−2.2	81	39
ENN A1	6	2.0	6	4.1	5	3.0	7	8.5	91	68
ENNA	6	−6.7	9	−16.8	12	−12.1	12	−9.9	89	33
ENNB	5	8.2	4	6.2	5	8.2	7	10.0	93	47
ENNB1	10	−10.4	9	0.4	12	−7.7	10	6.5	86	47
BEA	8	−5.7	7	3.2	6	−3.3	7	7.3	111	41
FB2	6	8.0	10	4.8	13	−1.7	14	−1.9	71	15
FB1	11	−1.7	6	0.7	10	0.5	7	1.6	77	17

Note: acceptance criteria: accuracy, ≥10 ng·mL^−1^: −20% to +10%. Within-day precision: RSD% < RSDmax with RSDmax for ≥10 to <100 ng·mL^−1^: <15%. Between-day precision: the RSD% < RSDmax with RSDmax 22.6% and 32% for the respective concentrations of 100 and 10 ng·mL^−1^, respectively.

**Table 5:** Validation results for linearity shown as the mean ± standard deviation of three curves across three different days of analysis (linear range, correlation coefficient (*r*) and goodness-of-fit coefficient (g)) and limit of quantification (LOQ) of 23 mycotoxins in dried blood spots of dried blood spots of pig whole blood extracted from an 8 mm disk.

Analyte	LOQ (ng·mL^−1^)	Linearity (*n* = 3 Different Days)		
		Linear Range (ng·mL^−1^)	*r* ± SD	g ± SD
ZEN	1.0	1–200	0.994 ± 0.002	12 ± 4
AZEL	1.0	1–200	0.996 ± 0.002	13 ± 4
AZAL	1.0	1–200	0.996 ± 0.001	16 ± 3
BZAL	0.5	0.5–200	0.995 ± 0.003	19 ± 2
BZEL	0.5	0.5–200	0.995 ± 0.003	16 ± 4
ZAN	1.0	1–200	0.998 ± 0.002	14 ± 4
TeA	1.0	1–200	0.996 ± 0.002	19 ± 1
AOH	2.0	2–200	0.998 ± 0.001	18 ± 1
AME	1.0	1–200	0.997 ± 0.002	17 ± 4
DON	1.0	1–200	0.992 ± 0.002	15± 3
DOM1	1.0	1–200	0.995 ± 0.001	15 ± 2
3/15ADON	0.5	0.5–200	0.994 ± 0.003	18 ± 1
T2	0.5	0.5–200	0.998 ± 0.001	16 ± 5
AFB1	1.0	1–200	0.993 ± 0.002	12 ± 3
AFM1	0.5	0.5–200	0.996 ± 0.003	13 ± 2
OTA	1.0	1–200	0.996 ± 0.001	14 ± 5
ENNA1	0.5	0.5–200	0.995 ± 0.002	15 ± 3
ENNA	0.5	0.5–200	0.997 ± 0.002	18 ± 2
ENNB	0.5	0.5–200	0.997 ± 0.001	12 ± 7
ENNB1	1	1–100	0.995 ± 0.003	10 ± 2
BEA	0.5	0.5–200	0.993 ± 0.001	15 ± 3
FB1	1.0	1–200	0.995 ± 0.002	17 ± 3
FB2	1.0	1–200	0.998 ± 0.001	11 ± 5

Note: SD, standard deviation; acceptance criteria: *r* ≥ 0.990 and g ≤ 20%.

**Table 6:** Validation results for between-day and within-day precision and accuracy of 23 mycotoxins in dried blood spots of pig whole blood extracted from an 8 mm disk.

Analyte	Within-day Precision and Accuracy (*n* = 6)				Between-day Precision and Accuracy (*n* = 3 × 3)			
	Theoretical Concentration 10 ng·mL^−1^		Theoretical Concentration 100 ng·mL^−1^		Theoretical Concentration 10 ng·mL^−1^		Theoretical Concentration 100 ng·mL^−1^	
	Precision (RSD %)	Accuracy (%)	Precision (RSD %)	Accuracy (%)	Precision (RSD %)	Accuracy (%)	Precision (RSD %)	Accuracy (%)
ZEN	6	−4.2	6	−5.0	7	−2.2	9	0.4
AZEL	8	0.1	4	−4.8	13	−3.5	14	−4.5
AZAL	8	−3.4	5	−12.5	13	−3.2	14	−4.1
BZAL	4	−2.6	6	−10.0	7	−4.1	9	−5.1
BZEL	7	6.1	4	−3.1	10	0.7	13	−3.4
ZAN	6	5.3	4	−0.3	7	2.6	13	1.0
TeA	6	−1.0	4	2.8	16	−8.8	8	−1.1
AOH	12	−16.3	10	−0.3	21	−9.9	11	−1.9
AME	14	−3.7	10	−6.0	27	−1.9	18	−10.1
DON	7	−1.2	4	−3.6	15	7.2	9	3.2
DOM1	6	6.0	5	−7.0	26	−11.5	10	−2.3
3/15ADON	7	−4.0	5	−10.7	10	−8.0	12	−8.0
T2	8	−9.5	6	−18.8	9	−4.3	17	−5.3
AFB1	5	−2.6	8	−6.0	8	−4.5	12	−6.0
AFM1	5	−9.7	9	−18.0	13	−8.7	14	−9.6
OTA	13	−3.0	6	−10.9	12	−8.2	10	−7.5
ENN A1	9	9.3	4	7.5	10	9.8	10	6.5
ENNA	8	7.0	8	−5.1	8	8.0	13	2.1
ENNB	6	6.4	10	−19.2	9	9.9	19	−9.0
ENNB1	7	9.2	8	9.8	7	8.9	15	5.1
BEA	1	7.1	5	−19.4	5	9.6	21	1.7
FB2	6	−16.8	6	−19.9	21	−7.0	20	−8.6
FB1	10	−4.9	3	−17.5	22	−7.5	17	−9.0

Note: acceptance criteria: accuracy, ≥10 ng·m^−1^: −20% to +10%. Within-day precision: RSD% < RSDmax with RSDmax ≥10 to <100 ng·mL^−1^: <15%. Between-day precision: the RSD% < RSDmax with RSDmax 22.6% and 32% for the respective concentrations of 100 and 10 ng·mL^−1^, respectively.

## Supplementary Materials

The following are available online at https://www.mdpi.com/2072-6651/11/9/541/s1, Figure S1: Example of a Whatman^®^ 903 protein saver card spotted with 60 µL of blood. Figure S2: Comparison of mycotoxin concentrations upon extraction of the entire blood spot and the 8 mm disk in DBS obtained after administration of (A) DON (36 µg·kg^−1^ BW) and (B) AFB1 (0.1 mg·kg^−1^ BW) to pigs (*n* = 2). Figure S3: Comparison of mycotoxin concentrations determined after LC-MS/MS analysis of dried blood spots and plasma samples obtained after a single intra-gastric bolus administration of deoxynivalenol (DON) (36 µg·kg^−1^ BW), aflatoxin B1 (AFB1) (0.1 mg·kg^−1^ BW) and zearalenone (ZEN) (3 mg·kg^−1^ BW) to pigs (*n* = 2). Figure S4: Comparison of mycotoxin concentrations determined after LC-MS/MS analysis of dried blood spots and plasma samples obtained after single intra-crop bolus administration of ochratoxin A (OTA) (0.25 mg·kg^−1^ BW), aflatoxin B1 (AFB1)(2 mg·kg^−1^ BW) and deoxynivalenol (DON)(0.5 mg·kg^−1^ BW) to broiler chickens (*n* = 2). Figure S5: Chromatograms of FB1 and DON in plasma and DBS of sows suffering from postpartum problems obtained in an exposure assessment study performed in 5 pig farms. (A and B) show the presence of DON (2.07 ng/mL) and FB1 (50 ng/mL), respectively in dried blood spots and (C and D) the presence of DON (<LOQ) and FB1 (not quantified) in plasma, respectively. Table S1: Overview of the parent mycotoxins and possible phase I and II metabolites included in the LC-MS/MS and LC-HRMS analysis described by Lauwers et al. [3]. Table S2: Overview of the structure of the parent mycotoxins and phase I metabolites included in the analysis.
