# Supplementary material for: Assessment of Dried Blood Spots for Multi-Mycotoxin Biomarker Analysis in Pigs and Broiler Chickens
Source: Toxins (Basel). 2019 Sep 18;11(9):541. doi: 10.3390/toxins11090541 (PMC6784198; doi:10.3390/toxins11090541)
Supplement: Supplementary file 1 [file toxins-11-00541-s001.pdf]

Supplementary Materials: Assessment of Dried Blood Spots for Multi-Mycotoxin Biomarker Analysis in Pigs and Broiler Chickens

Marianne Lauwers, Siska Croubels, Siegrid De Baere, Milena Sevastiyanova

Eva Maria Romera Sierra, Ben Letor, Christos Gougoulias and Mathias Devreese

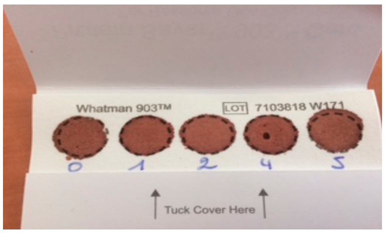

Figure S1. Example of a Whatman® 903 protein saver card spotted with 60 µL of blood.

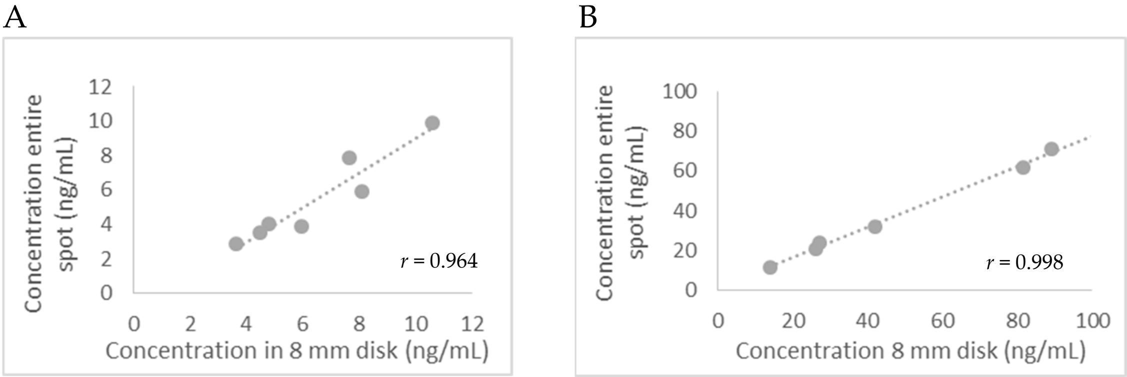

Figure S2. Comparison of mycotoxin concentrations upon extraction of the entire blood spot and the 8 mm disk in DBS obtained after administration of (**A**) DON (36 µg·kg^{-1} BW) and (**B**) AFB1 (0.1 mg·kg^{-1} BW) to pigs (n = 2).

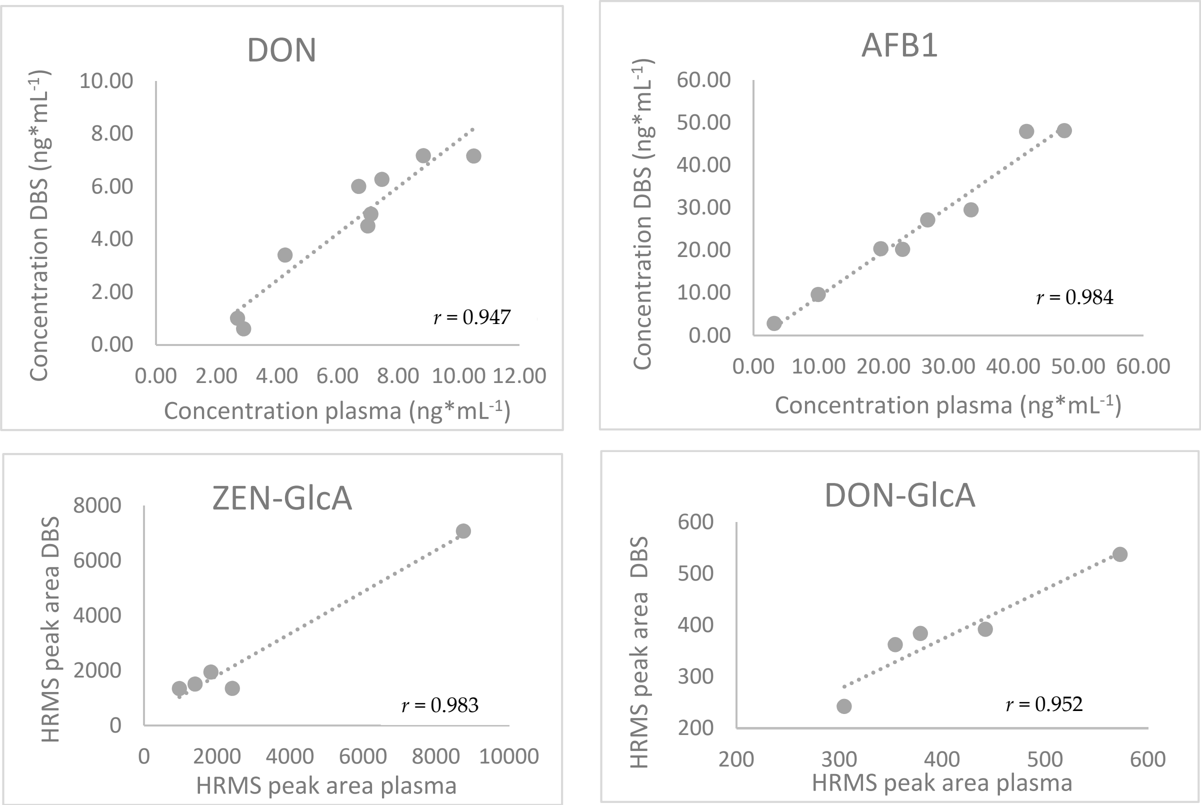

Figure S3. Comparison of mycotoxin concentrations determined after LC-MS/MS analysis of dried blood spots and plasma samples obtained after a single intra-gastric bolus administration of deoxynivalenol (DON) (36 μg·kg^{-1} BW), aflatoxin B1 (AFB1) (0.1 mg·kg^{-1} BW) and zearalenone (ZEN) (3 mg·kg^{-1} BW) to pigs ($n = 2$).

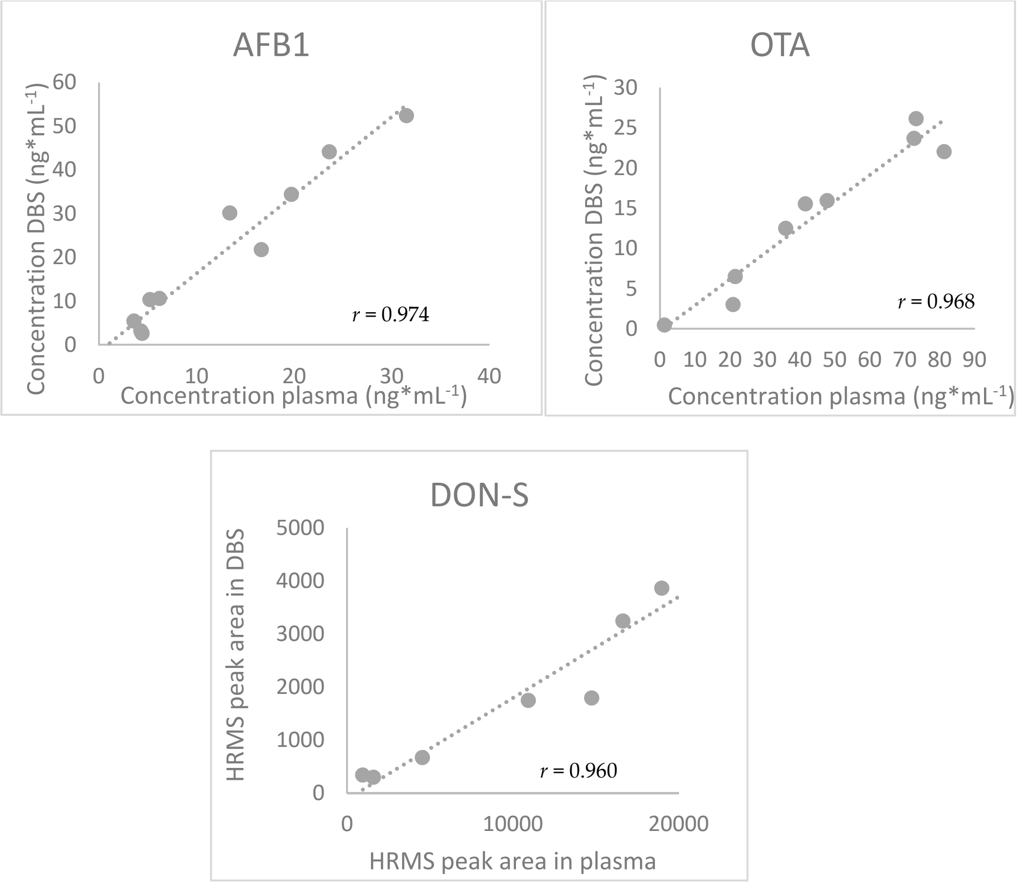

Figure S4. Comparison of mycotoxin concentrations determined after LC-MS/MS analysis of dried blood spots and plasma samples obtained after single intra-crop bolus administration of ochratoxin A (OTA) (0.25 mg·kg^{-1} BW), aflatoxin B1 (AFB1)(2 mg·kg^{-1} BW) and deoxynivalenol (DON)(0.5 mg·kg^{-1} BW) to broiler chickens (n = 2).

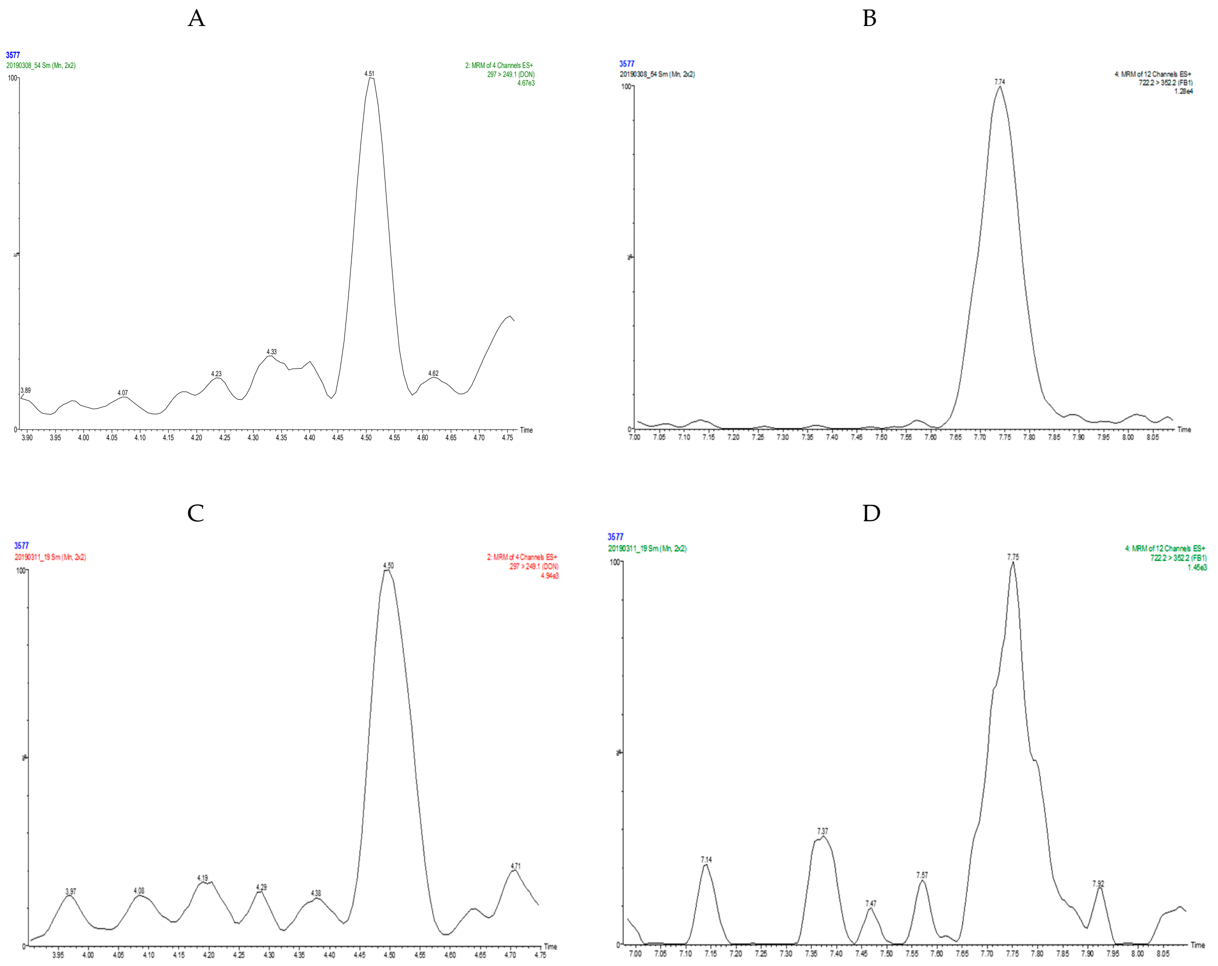

Figure S5. Chromatograms of FB1 and DON in plasma and DBS of sows suffering from postpartum problems obtained in an exposure assessment study performed in 5 pig farms. (**A** and **B**) show the presence of DON (2.07 ng/mL) and FB1 (50 ng/mL), respectively in dried blood spots and (**C** and **D)** the presence of DON (<LOQ) and FB1 (not quantified) in plasma, respectively.

Table S1. Overview of the parent mycotoxins and possible phase I and II metabolites included in the LC-MS/MS and LC-HRMS analysis described by Lauwers et al [3].

**Name**	**Name**
Deoxynivalenol	$^{13}C_{15}$-Deoxynivalenol
De-epoxy-deoxynivalenol	$^{13}C_{17}$-Aflatoxin B1
3/15-acetyldeoxynivalenol	$^{13}C_{20}$-Ochratoxin A
T2-toxin	$^{13}C_{24}$-T2-toxin
HT2-toxin	$^{13}C_{34}$-Fumonisin B1
Aflatoxin B1	$^{15}N_{3}$-Enniatin B
Aflatoxicol	$^{13}C_{6}{}^{15}N$-Tenuazonic acid
Aflatoxin M1	$^{13}C_{18}$-Zearalenone
Ochratoxin A	Deoxynivalenol-glucuronide
Enniatin A1	De-epoxy-deoxynivalenol glucuronide
Enniatin A	Deoxynivalenol-sulphate
Enniatin B	Deoxynivalenol-di-sulphate
Enniatin B1	3/15-acetyl-deoxynivalenol-sulphate
Beauvericin	Ochratoxin alfa
Fumonisin B1	Zearalenone-glucuronide

Fumonisin B2	Zearalenone-di-glucuronide
Tenuazonic acid	α/β-zearalenol- or zearalanone-glucuronide
Alternariol	Zearalenone-sulphate
Alternariol methyl ether	α/β-zearalenol- or zearalanone-sulphate
Zearalenone	α/β-zearalenol-sulphate
Zearalanone	Tenuazonic acid-sulphate
α-Zearalenol	Alternariol-sulphate
α-Zearalanol	Alternariol-methyl ether sulphate
β-Zearalanol	
β-Zearalenol	

Table S2. Overview of the structure of the parent mycotoxins and phase I metabolites included in the analysis.

Analyte	Chemical Structure	Analyte	Chemical Structure	Analyte	Chemical Structure	Analyte	Chemical Structure
ZEN	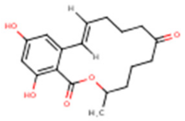 $C_{18}H_{22}O_5$	T2	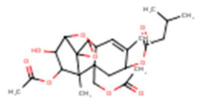 $C_{24}H_{34}O_9$	TEA	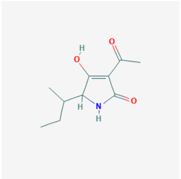 $C_{10}H_{15}NO_3$	ENNB	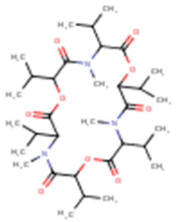 $C_{33}H_{57}N_3O_9$
AZEL	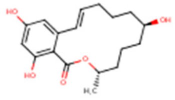 $C_{18}H_{24}O_5$	AFB1	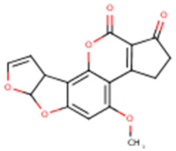 $C_{17}H_{12}O_6$	AOH	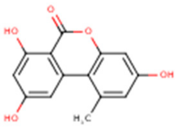 $C_{14}H_{10}O_5$	ENNB1	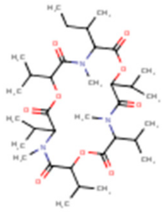 $C_{34}H_{59}N_3O_9$
AZAL	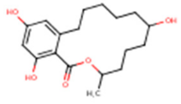 $C_{18}H_{26}O_5$	AFM1	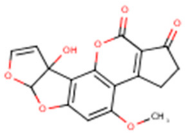 $C_{17}H_{12}O_7$	AME	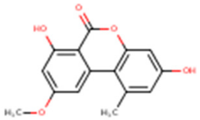 $C_{15}H_{12}O_5$	BEA	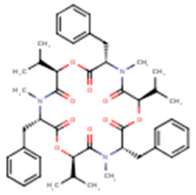 $C_{45}H_{57}N_3O_9$
BZAL	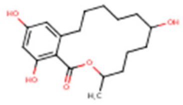 $C_{18}H_{26}O_5$	OTA	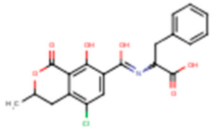 $C_{20}H_{18}ClNO_6$	DON	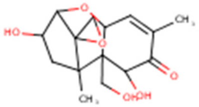 $C_{15}H_{20}O_6$	FB1	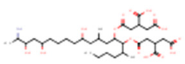 $C_{34}H_{59}NO_{15}$

BZEL	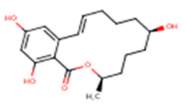 $C_{18}H_{24}O_5$	ENN A1	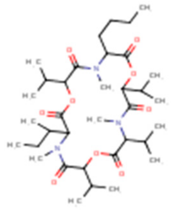 $C_{35}H_{61}N_3O_9$	DOM-1	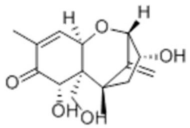 $C_{15}H_{20}O_5$	FB2	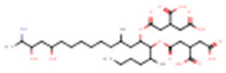 $C_{34}H_{59}NO_{14}$
ZAN	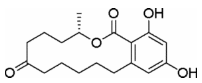 $C_{18}H_{24}O_5$	ENNA	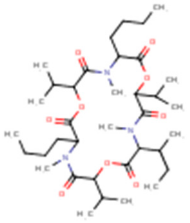 $C_{36}H_{63}N_3O_9$	15-ADON	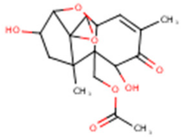 $C_{17}H_{22}O_7$	3-ADON	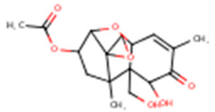 $C_{17}H_{22}O_7$
